# Effect of the carbohydrate counting method on glycemic control in patients with type 1 diabetes

**DOI:** 10.1186/1758-5996-2-54

**Published:** 2010-08-17

**Authors:** Viviane M Dias, Juliana A Pandini, Raquel R Nunes, Sandro LM Sperandei, Emilson S Portella, Roberta A Cobas, Marília de B Gomes

**Affiliations:** 1 Pedro Ernesto University Hospital (HUPE). Av. 28 de Setembro, n. 77, Vila Isabel - Rio de Janeiro - CEP: 20551-030 - RJ - Brazil

## Abstract

**Background:**

The importance of achieving and maintaining an appropriate metabolic control in patients with type 1 diabetes mellitus (DM1) has been established in many studies aiming to prevent the development of chronic complications. The carbohydrate counting method can be recommended as an additional tool in the nutritional treatment of diabetes, allowing patients with DM1 to have more flexible food choices. This study aimed to evaluate the influence of nutrition intervention and the use of multiple short-acting insulin according to the carbohydrate counting method on clinical and metabolic control in patients with DM1.

**Methods:**

Our sample consisted of 51 patients with DM1, 32 females, aged 25.3 ± 1.55 years. A protocol of nutritional status evaluation was applied and laboratory analysis was performed at baseline and after a three-month intervention. After the analysis of the food records, a balanced diet was prescribed using the carbohydrate counting method, and short-acting insulin was prescribed based on the total amount of carbohydrate per meal (1 unit per 15 g of carbohydrate).

**Results:**

A significant decrease in A1c levels was observed from baseline to the three-month evaluation after the intervention (10.40 ± 0.33% and 9.52 ± 0.32%, respectively, p = 0.000). It was observed an increase in daily insulin dose after the intervention (0.99 ± 0.65 IU/Kg and 1.05 ± 0.05 IU/Kg, respectively, p = 0.003). No significant differences were found regarding anthropometric evaluation (BMI, waist, hip or abdominal circumferences and waist to hip ratio) after the intervention period.

**Conclusions:**

The use of short-acting insulin based on the carbohydrate counting method after a short period of time resulted in a significant improvement of the glycemic control in patients with DM1 with no changes in body weight despite increases in the total daily insulin doses.

## Introduction

Diabetes mellitus (DM) is considered a major public health issue because of its increasing prevalence and high morbidity and mortality. Recent data from the World Health Organization (WHO) estimate that there will be 333 million people with DM in the year of 2030, and 11 million of these will be Brazilian people [1]. In Brazil, the average prevalence of DM was 7.6% in people aged 30-69 years in the 1980's and 30-50% were undiagnosed cases [2]. Approximately 5-10% of people with DM present type 1 diabetes (DM1).

Hyperglycemia is directly related to the development and progression of microvascular complications in patients with DM [3]. The effect of intensive insulin treatment of the patients aiming to reduce A1c levels has been shown to reduce the risk of diabetes microvascular complications [4-6]. In addition, a study conducted by Moss *et al*., with individuals with DM1 followed up for 10 years, showed positive association between glycated hemoglobin levels and coronary artery disease [7]. Based on these facts, the American Diabetes Association (ADA) [8] emphasizes the importance of a good glycemic control to prevent chronic diabetes complications and suggests that changes in the diet composition of patients with DM are relevant strategies to achieve an appropriate metabolic control.

One available strategy is the carbohydrate counting method that allows patients to have a more flexible food choice. This method has been used since 1935 in Europe and was adopted by The Diabetes Control and Complication Trial (DCCT). The ADA, in 1994, recommended this method as an additional tool in the nutritional treatment of DM. It started to be used in Brazil by few professionals in 1997, but nowadays many groups use this method in a systematic way [9].

This study aimed to evaluate the influence of nutrition intervention on the clinical and metabolic parameters in patients with DM1, using multiple dose of short-acting insulin according to the carbohydrate counting method.

## Subjects and Methods

This study was conducted in outpatients with DM1 followed up at Pedro Ernesto University Hospital [*Hospital Universitário Pedro Ernesto*] (HUPE), diagnosed according to the ADA criteria [10], aged 10-60 years. The exclusion criteria were illiteracy, diabetic nephropathy or retinopathy, pregnancy and mobility impairment. The study protocol was approved by the Research Ethics Committee of Pedro Ernesto University Hospital and a consent form was signed by the participants.

Patients included in the study followed their normal dietary prescription without having received any previous guidance on the carbohydrate counting of foods in the diet.

Patients were evaluated at baseline and after a three-month period of follow-up. At the first meeting, a protocol of nutritional status evaluation was applied and blood samples were collected. The protocol consisted of personal data, previous pathology history, family history, information on type and dose of insulin used. The nutrition evaluation consisted of anthropometric, biochemical and dietary information.

The anthropometric parameters included measurement of body weight (BW), stature, abdominal circumference, and hip and waist circumference. A previously tared Filizola^® ^plataform scale, with 0.1 kg precision and maximum capacity of 150 kg, was used to determine BW. Before BW evaluation, shoes, all excessive clothes and any object that could interfere with the precise BW determination were removed. During this procedure, patients maintained the feet at the centre of the scale, the body erected with the weight distributed equally between the two feet, without making any sort of movements, with the arms along the body and the back turned to the display screen. Stature was determined using a Tonelli & Gomes^® ^stadiometer with graduation of 0.1 cm, tested and approved by the pediatric endocrinology unit of the Federal University of Paraná. In order to avoid bias, patients were without shoes, in orthostatic position. During the evaluation, patients breathed in profoundly while the examiner put the horizontal stem of the stadiometer in the appropriate position (the highest point of the head). Based on BW and stature data, body mass index (BMI) was calculated and used to classify adults according to the cut-offs proposed by the World Health Organization (WHO, 1998) [11] as underweight (BMI under 18.5 kg/m^2^), normal range (BMI between 18.5 and 24.9 kg/m^2^), pre-obese (BMI between 25.0 and 29.9 kg/m^2^), obese class I (BMI between 30.0 and 34.9 kg/m^2^), obese class II (BMI between 35.0 and 39.9 kg/m^2^) and obese class III (BMI equal or above 40.0 kg/m^2^). Children less than 10 years old did not participate in this study. The BMI percentiles for teenagers were those presented by Monteiro and Conde [12].

Abdominal circumference (AC) was measured at the level of the umbilicus, and waist circumference (WC) was measured at the medium point between the last inferior rib and the iliac crest. Hip circumference (HC) was measured in the widest point of the greater trochanters. Patients stayed erected maintaining relaxed the region that would be measured and without clothes. An inelastic metric tape was used to obtain the measurements mentioned above. Waist-to-hip ratio (WHR) was calculated, dividing waist circumference by hip circumference (both measurements in cm).

A blood collection sample was obtained after a minimum of 10 hours fasting. The colorimetric enzymatic method was used to determine plasma glycemia, total cholesterol, HDL-cholesterol and triglyceride levels. LDL-cholesterol was calculated by the Friedwald formula in patients with triglycerides < 400 mg/dl [13].

Glycated hemoglobin analysis was performed by the HLPC method (Hitachi Hech-9000 V.R.) with normal range from 4.4 to 6.4%. Postprandial glycemia was evaluated two hours after the usual breakfast.

In order to evaluate dietary intake, patients filled out a three-day food record (three consecutive days with one day of the weekend), only at the baseline, in which they described information about food intake, meal time and places where meals were eaten. Complementary information included addition of salt to the meals, intake of refined sugar, oil, sauces, diet or light products, industrialized food and the way meals were prepared. This method is known as estimated record of food intake since foods are estimated using measurement conversion tables. Portions were determined in household measures (spoons, cups, shallow or deep plates).

Records were made by the patient after being previously instructed and were reviewed by the dietitian who analyzed the diet macronutrients centesimal composition using national tables of food composition [14,15]. After the careful analysis of the records, a balanced diet was prescribed based on the carbohydrate counting method considering the food habits of the patients and adapted for them. It was also given a substitution food list.

Insulin dose was adjusted to the amount of carbohydrate in each meal based on the following relationship: one unit of short-acting human insulin for every 15 g of ingested carbohydrate. All participants used multiple daily injections of short-acting insulin at meals with NPH as basal and at night. The short-acting insulin dose was prescribed before breakfast taking into account breakfast and the mid-morning snack, the insulin dose prescribed before lunch covered lunch and the mid-afternoon snack, and the insulin dose prescribed before dinner covered dinner and supper. No participant used self-blood glucose monitoring during the study period.

Participants were followed up with visits to the dietitian every 15 days, where dietary history was made to verify adherence to the diet of carbohydrate counting. We did not use any other tool for nutritional assessment during the three-month follow-up period.

Data were collected and analyzed by the Statistical Package for the Social Sciences (SPSS), version 13.0 for Windows. All variables were tested for normality, and data were presented as mean ± standard deviation. Paired t-test was used to compare baseline, data were collected after the three-month period, and p-value < 0.05 was considered statistically significant.

## Results

Initially, 55 patients aged 25.3 ± 1.55 years took part in the study. Four patients were excluded because they did not attend the follow-up. Fifty-one patients with DM1 completed the study, 37% males and 63% females, with duration of DM of 11.31 ± 1.09 years.

**Figure 1:**
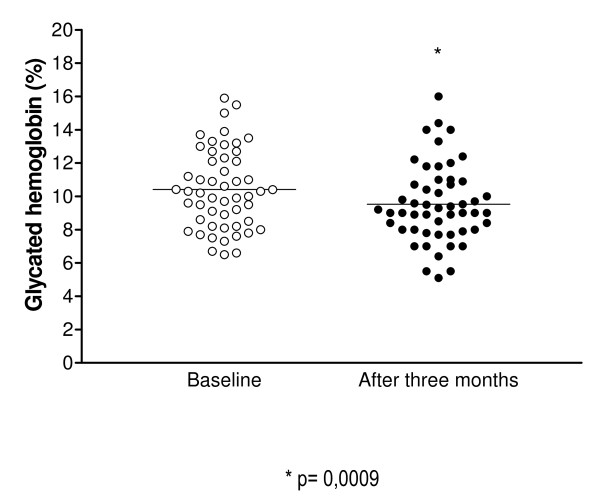
**A1c values at baseline and after the intervention**.

A significant decrease in A1c levels was observed from baseline to the three-month evaluation (10.40 ± 0.33% and 9.52 ± 0.32%, respectively, p = 0.0009). These data are shown in Figure 1.

A significant reduction in A1c levels was observed in 38 (74%) patients. Conversely, 11 diabetics (21.5%) presented an increase in this value, whereas two patients (3.9%) maintained the same baseline values. The proportion of patients that presented A1c levels < 7% at the baseline and after the intervention did not differ [3 (5.9%) vs. 4 (7.5%) patients].

Additional biochemical analysis did not reveal any significant difference. Baseline and post-treatment data are described in detail in Tables 1 and 2, separated by gender.

**Table 1:** Clinical and laboratorial data of sample before and after the therapeutic intervention in all patients studied.

Variables	Baseline	3 months	Significance level (p)
n (number)	51	51	
Gender(M/F)	19/32	19/32	
Age(years)	25,3 ± 1,55	25,3 ± 1,55	
Duration of diabetes (years)	11,31 ± 1,09	11,31 ± 1,09	
BMI (kg/m^2^)	22,87 ± 0,42	23,20 ± 0,47	0,51
sBP (mmHg)	111,3 ± 1,96 (180,0- 90,0)	111,24 ± 1,76 (180,0- 90,0)	0,81
sDP(mmhg)	70,24 ± 1,14 (90,0- 50,0)	90,03 ± 10,12 (100,0- 50,0)	0,32
A1c (%)	10,40 ± 0,33	9,52 ± 0,32	0,0009
A1c < 7% (n pacientes)	3	4	
Cholesterol(mg/dl)	174,88 ± 5,58	169,94 ± 4,58	0,23
HDL-Cholesterol(mg/dl) LDL- Cholesterol(mg/dl)	53,84 ± 1,94 210,58 ± 6,05	53,40 ± 1,91 202,72 ± 4,86	0,79 0,11
Triglycerides (mg/d)	93,23 ± 7,60	104,63 ± 10,29	0,07
Fasting blood sugar FBS (mg/dl)	180,00 ± 21,22	168,82 ± 14,79	0,73
Postprandial glycemia (mg/dl)	256,78 ± 12,82	243,39 ± 15,92	0,46
Insulin dose/kg	0,99 ± 0,65	1.05 ± 0.05	0,00
NPH insulin dose	55,08 ± 22,34	55,18 ± 21,10	0,89
Rapid insulin dose	4,42 ± 8,72	11,73 ± 7,04	0,00

**Table 2:** Clinical and laboratorial data of sample before and after the therapeutic intervention, separated by gender.

Variables	Men			Women		
	Baseline	3 months	Significance Level (p)	Baseline	3 months	Significance level (p)
n (number)	19	19		32	32	
Duration of diabetes (years)	9,24 ± 1,41	9,24 ± 1,41	0,124	12,34 ± 1,48	12,34 ± 1,48	0,167
BMI (kg/m^2^)	22,55 ± 0,62	22,88 ± 0,65	0,37	23,0 ± 0,57	23,0 ± 0,65	0,47
sBP (mmHg)	120 ± 0,75	110 ± 1,78	0,58	100,82 ± 0,29	110 ± 0,38	0,33
sDP(mmHg)	70,67 ± 1,23	70,75 ± 1,28		60,95 ± 1,04	90,59 ± 12,40	
A1c (%)	10,28 ± 0,55	9,30 ± 0,50	0,01	10,00 ± 2,42	9,65 ± 2,45	0,00
Cholesterol(mg/dl)	159,63 ± 6,37	163,73 ± 5,70	0,164	183 ± 7,69	179,56	0,48
HDL- Cholesterol(mg/dl)	50,63 ± 3,63	49,18 ± 4,07	0,46	55,7 ± 2,19	55,90 ± 1,77	0,92
LDL- Cholesterol (mg/dl)	193,71 ± 7,86	184,41 ± 6,13	0,13	220,59 ± 8,00	213,59 ± 6,13	0,32
Triglycerides (mg/dl)	89,73 ± 11,18	96,68 ± 14,25	0,49	95,31 ± 10,2	109 ± 14,15	0,09
Fasting blood sugar- (mg/dl)	153,84 ± 28,37	153,84 ± 28,37	0,74	200,34 ± 20,32	171,12 ± 18,85	0,25
Postprandial glycemia (mg/dl)	213,05 ± 16,83	231,47 ± 20,64	0,46	282 ± 16,32	250,46 ± 22,44	0,19
Insulin dose/kg	0,89 ± 0,31	1,05 ± 0,31	0,00	1,07 ± 0,53	1,13 ± 0,49	0,10
NPH insulin dose	55,22 ± 19,90	56,89 ± 18,52	0,16	55,00 ± 23,91	54,22 ± 22,65	0,40
Rapid insulin dose	3,65 ± 5,30	3,12 ± 2,34	0,67	4,84 ± 10,17	12,13 ± 7,77	0,00

It was observed an increase in daily insulin dose/kg of BW after the intervention, when compared to the baseline values (1.05 ± 0.05 vs. 0.99 ± 0.65, respectively, p = 0.003). The short-acting insulin increase occurred in all doses, at breakfast from 2.25 ± 5.12 to 3.81 ± 4.53 (p = 0.00), at lunch from 0.92 ± 2.05 to 4.13 ± 2.05 (p = 0.00), and at dinner from 2.28 ± 1.89 to 3.32 ± 2.50 (p = 0.00). No significant differences were observed in doses of NPH insulin at breakfast from 34.06 ± 12.06 to 34.20 ± 12.19 (p = 0.64), at lunch from 2.38 ± 5.83 to 1.82 ± 4.33 (p = 0.28), and at diner from 18.64 ± 12.18 to 19.16 ± 11.41 (p = 0.42).

No significant differences were found regarding the anthropometric evaluation (BMI, WC, HC, AC and WHR) after the intervention period. The values found at baseline and three months after the therapeutic intervention were, respectively: BMI, 22.87 ± 0.42 vs. 23.20 ± 0.47 Kg/m^2 ^(p = 0.51); WC 75.33 ± 1.04 vs. 74.98 ± 1.09 cm (p = 0.509); AC, 79.28 ± 1.23 vs. 79.40 ± 1.17 cm (p = 0.731); HC, 94.02 ± 1.15 vs. 94.35 ± 1.27 cm (p = 0.325) and WHR, 0.79 ± 0.0 vs. 0.78 ± 0.61 (p = 0.161).

**Figure 2:**
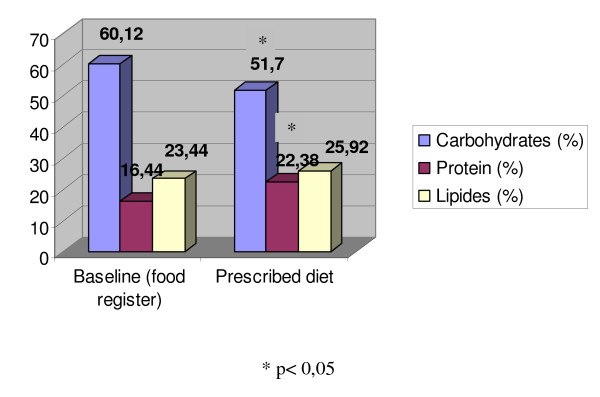
**Nutritional composition of diet of Carbohydrate counting and food register**.

Average calorie intake observed during the three days of food record was higher than the average calorie intake proposed by the prescribed diet based on carbohydrate counting. There was a reduction in the carbohydrate percentage in food record when compared to the amount recommended in the prescribed diet. On the other hand, protein intake in food record surpassed the amount in the prescribed diet. The comparison between total calorie intake and the percentage of macronutrient distribution in the three days of food record with the values recommended by the prescribed diet are described in Table 3 and Figure 2.

**Table 3:** Caloric intake and nutritional composition of diet of Carbohydrate counting and food register.

	Baseline (base don food register)*	Prescribed diet	p
Kcal	1694,33 ± 517,32	1588,49 ± 41,24	0,43

Carbohydrates (%)	60,12 ± 21,75	51,70 ± 6,91	0,004

Protein (%)	16,44 ± 12,23	22,38 ± 3,01	0,001

Lipides (%)	23,44 ± 13,83	25,92 ± 4,57	0,20

* without intervention for carbohydrate counting method

## Discussion

Carbohydrate counting is not a diet but a method that emphasizes glycemic control based on the use of multiple doses of short-acting insulin according to carbohydrate intake in a meal [16].

The goal of the therapeutic plan of our study is the appropriate adherence to the treatment aiming a metabolic control.

The A1c levels were reduced significantly after the study intervention, although they did not achieve the established values for a good glycemic control. It is known that a 1% fall in A1c results in significant decrease in microvascular complications, as described by DCCT [4] which compared the intensive treatment to the conventional treatment. This reduction can yield a more suitable clinical and metabolic control of the diabetes and, consequently, improve the lifestyle of these patients [17]. The significant decrease in A1c levels in our study allows us to conclude that adherence to the diet was adequate.

We did not observe differences regarding fasting and postprandial glycemia, mainly because both of them represent isolated values and are highly influenced by countless factors, and therefore are not good parameters to evaluate glycemic control in DM1 owing to its great variability [18].

No weight gain was observed during the intervention period. Our study did not focus on intensive treatment because self-monitoring blood glucose was not used. However, weight gain could stem from the multiple-dose insulin therapy [19]. Although patients had an increase in the average of some anthropometric data during intervention, none of them was significant, due to the fact that the prescribed diet aimed at health habits and was in accordance with the macronutrient recommendations made by the Brazilian Society of Diabetes in 2007[20].

The increase in the insulin dose can be explained by the multiple peaks of rapid-acting insulin schemes. The administration of insulin in an intensive treatment should seek to reproduce as close as possible the normal physiologic insulin secretion, resulting in increase in the number of peaks and in the units of insulin/kg BW. Intensive insulin treatment, although beneficial in decreasing the risk of diabetic complications, can result in weight gain [21] and its consequences, such as hypertension and a more atherogenic lipid profile.

Considering the great number of patients with DM1 who cannot afford intensive insulin therapy, including patients of our sample, and that the distribution of glycemic self-monitoring device by DATASUS (Data Processing Company of the Unified Health System) [*Empresa de Processamento de Dados do Sistema Único de Saúde*] is irregular, the scheme proposed in this study was multiple pre-adjusted doses of insulin based on the amount of ingested carbohydrate. Even though it is not the ideal, it can result in positive observations, such as the reduction in A1c levels, as observed in our study. Recently, Franco & Costa (2005) reported that only a minority of patients with DM1 use home self-monitoring of glycemia [19].

There are many barriers for the use of glycemic self-monitoring, such as the high cost and the physical and psychological discomfort due to factors such as the blood collection by finger prick, the technique misunderstanding by the patient or by the professionals, the time available for the performance of the test, the interpretation of individual data at different periods so as to make the adequate adjustment of insulin doses according to dietary intake [22].

Comparing the prescribed diet with the centesimal analysis of food record, the distribution of macronutrients in the prescribed diet was within the recommended range for diabetics [23]. The prescribed diet preferred complex carbohydrate, low glycemic index food, low fat food and high fiber food, characterizing health food habits. According to Brand-Miller *et al*. (2002), high glycemic index food is related to hyperphagia and, consequently, obesity [23].

Patients from our study used a mix of NPH and regular insulin, the most used type of insulin in our country. This scheme is not usually reported in the literature in studies on glycemic control, which use more frequently insulin analogues, such as insulin lispro, insulin glusiline, ultra slow regular insulin and insulin aspart [24-27].

Considering the limitations of our study, it is noteworthy to state that improvement of glycemic control was not sufficient for achieving appropriate control (A1c < 7%), possibly because of the short period of intervention and the high levels of A1c at baseline. Additional time of follow-up could determine if this improvement of glycemic control would persist or stabilize. The absence of a control group was also a limitation of our study.

A second and significant limitation of this study is related to the fact that the monitoring of blood glucose was not carried out, so we had no data to characterize hypoglycemia, which is a common intercurrence in patients with multiple needle sticks and carbohydrate counting. Such data would certainly enrich our results.

## Conclusion

The intervention through the carbohydrate counting method produced a significant improvement of glycemic control in type 1 diabetic patients, even though it was not sufficient to reach an adequate glycemic control. Our results allow us to conclude that it is possible to use the carbohydrate counting method successfully without home self-monitoring. This fact should be considered in developing countries like Brazil where a good metabolic control is a difficult goal to be reached because of socioeconomic factors such as expensive blood tests strips for monitoring blood glucose and irregular supply of oral tablets and insulin by the national health care system.

## Competing interests

The authors declare that they have no competing interests.

## Authors' contributions

VMD, substantial contribution to the conception and design of the study, and data acquisition; JAP and RRN, contributions to data acquisition; SS, responsible for the analyses and data interpretation; ESP, involved in drafting the manuscript and revising it critically for important intellectual content; RAC, medical doctor responsible for ambulatory type 1 diabetic patients; MBG, involved in drafting the manuscript and revising it critically for important intellectual content, giving the final approval of the version to be published. All authors read and approved the final manuscript.

## Authors' information

**VMD and JAP**, dietitians with a master degree; trainees for CLINEX-UERJ;

**RRN**, dietitian with a master degree; post-graduate student of Medical Science - UERJ;

**SLMS**, doctoral student of Computational Biology and Systems - FIOCRUZ;

**ESP**, professor in the Nutrition Institute of the University of Rio de Janeiro State;

**RAC**, medical specialist in endocrinology, doctoral student at CLINEX-UERJ;

**MBG**, associate professor of the University of Rio de Janeiro State and coordinator of the discipline Diabetes and Metabology.
